# Supplementary material for: The CONSTANS flowering complex controls the protective response of photosynthesis in the green alga Chlamydomonas
Source: Nat Commun. 2019 Sep 10;10:4099. doi: 10.1038/s41467-019-11989-x (PMC6736836; doi:10.1038/s41467-019-11989-x)
Supplement: Supplementary file 1 — Supplementary Information [file 41467_2019_11989_MOESM1_ESM.pdf]

Supplementary Information

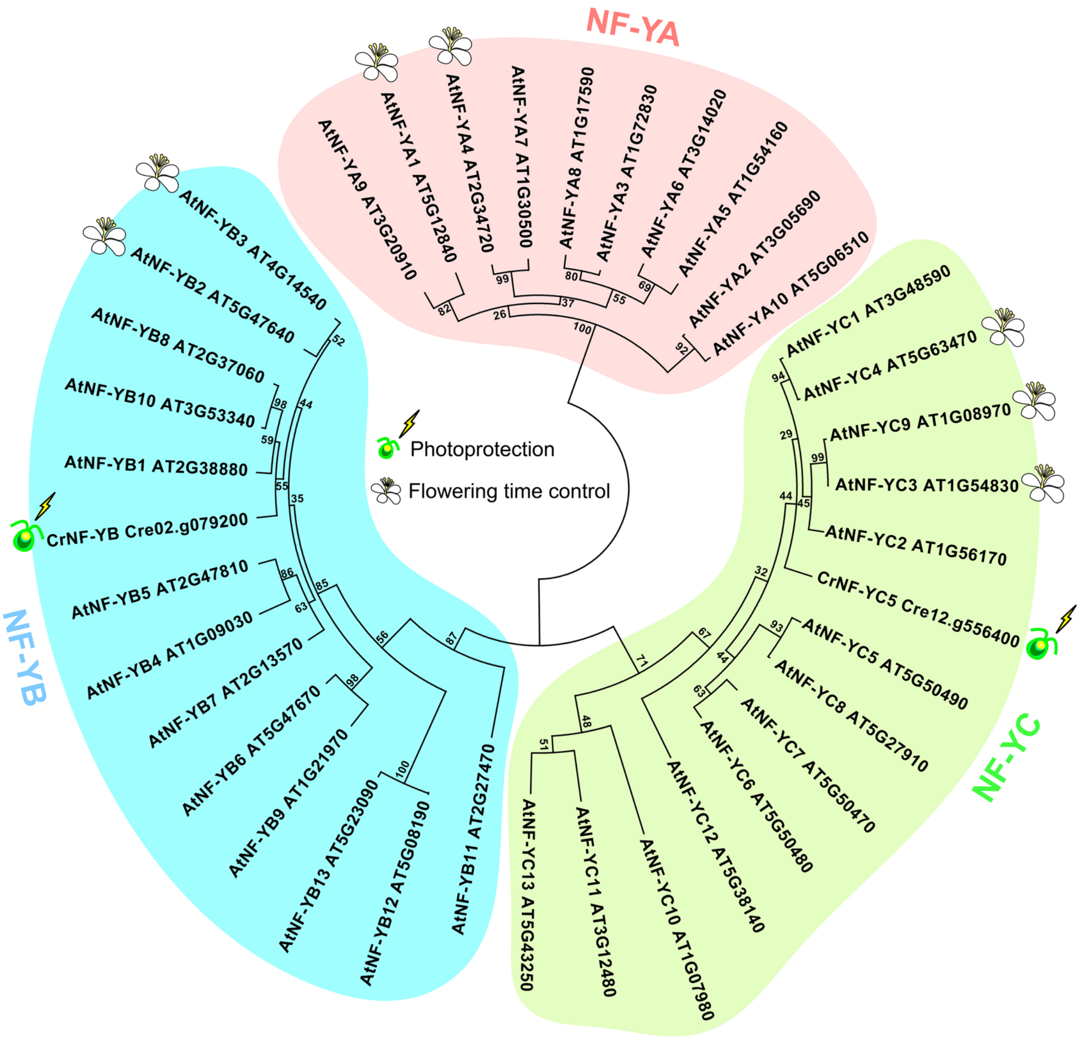

Supplementary Figure 1. Phylogenetic tree of NF-Y proteins from *A. thaliana* and *C. reinhardtii*. The phylogenetic tree represents the relationship between protein sequences of NF-YA from *A. thaliana* (AtNF-YA1–10) and NF-YB and NFYC from *A. thaliana* (AtNF-YB1–13 and AtNF-YC1–13) and *C. reinhardtii* (CrNF-YB and CrNF-YC). Proteins are products of the genes as labeled. The tree is drawn to relative scale, and branches are labeled with bootstrap values.

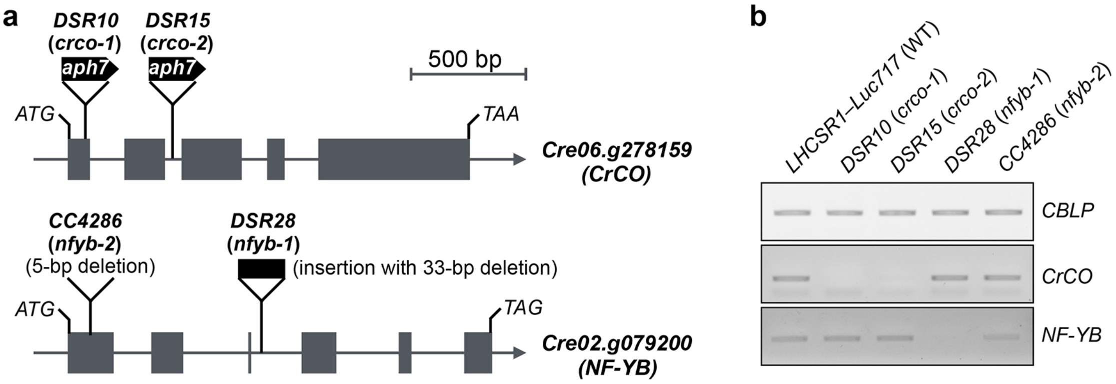

Supplementary Figure 2. Characterization of the mutations in *CrCO* and *NF-YB*.
(a) Schematic diagrams of the gene structure (chromosome) of *CONSTANS* (*Cre06.g278159*, *CrCO*) and *NUCLEAR TRANSCRIPTION FACTOR YB* (*Cre02.g089200*, *NFYB*). Translation start and stop codons, the position and orientation of the inserted *aph7* tags, the position of the deleted nucleotides, and the position at which unknown nucleotides were inserted in the mutants. The *CC4286* and *DSR28* strains are described in detail in Methods. (b) Total RNA extracted from mutants was analyzed by RT-PCR. *CBLP* signals are shown as the loading control.

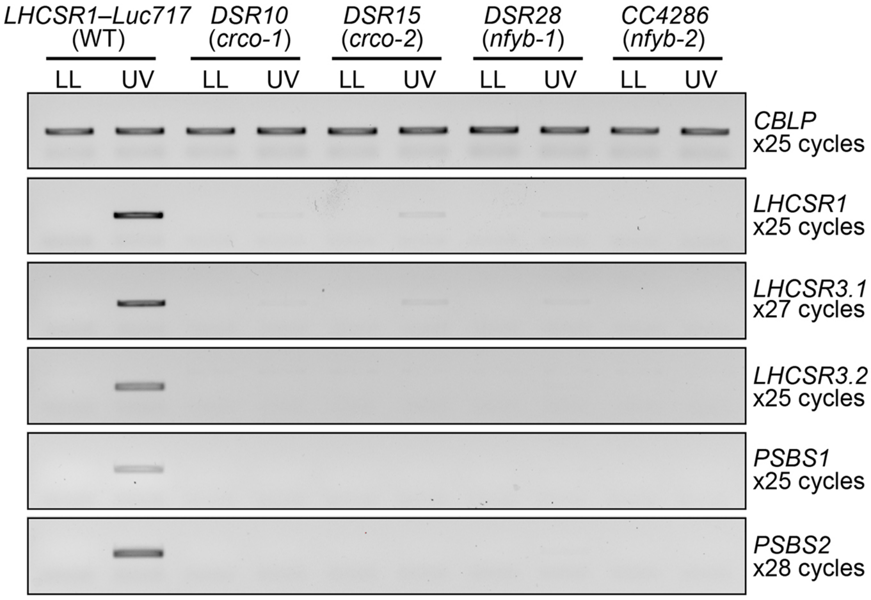

Supplementary Figure 3. Expression of the photoprotective genes in the *DSR* and *CC4286* mutants.

RT-PCR analysis of UV-inducible photoprotective genes (*LHCSR1*, *LHCSR3.1*, *LHCSR3.2*, *PSBS1*, and *PSBS2*) in control (*LHCSR1–Luc717*) and mutant (*DSR* and *CC4286*) *Chlamydomonas reinhardtii* strains before and after cells were subjected to 1 h of UV treatment. *CBLP* signals are shown as the loading control.

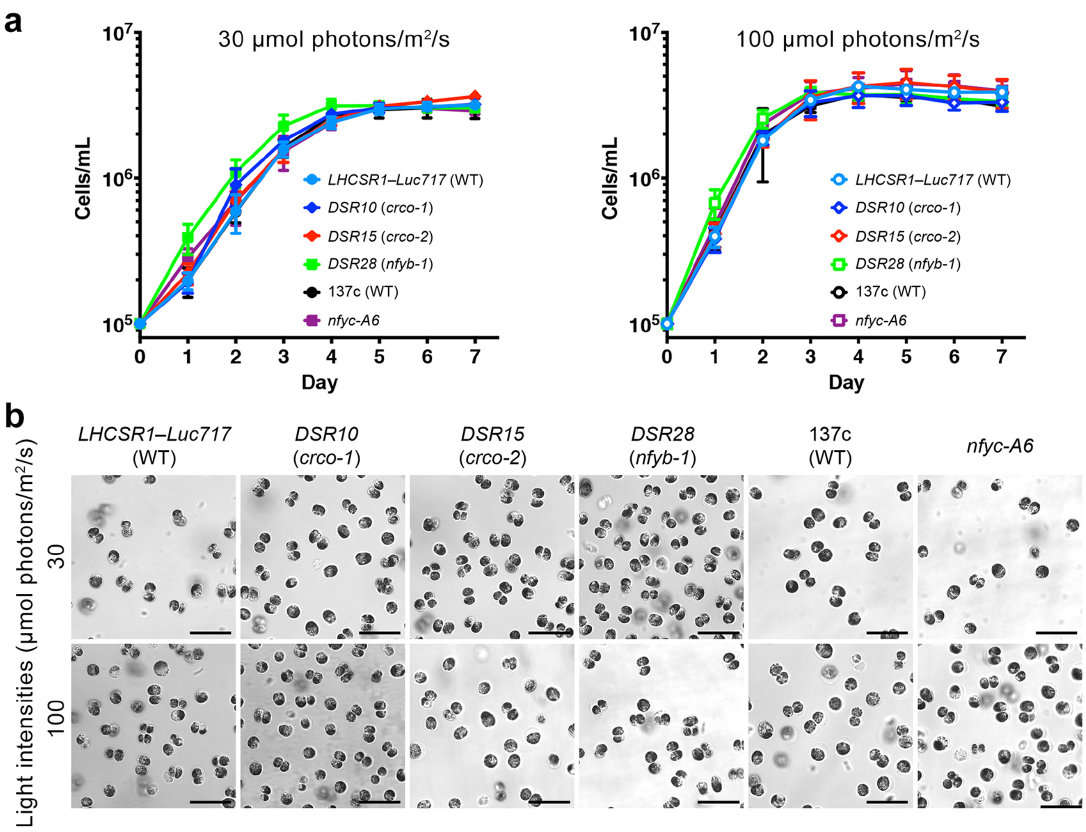

Supplementary Figure 4. Growth and cell shape of *CONSTANS* and *NF-Y* mutants under two different light intensities.

(a) Cell growth under 30 μmol photons m^{-2} s^{-1} or 100 μmol photons m^{-2} s^{-1} of white fluorescent light. (n = 4 biological replicates; mean ± s.d.). (b) Bright-field microscopy images of WT and mutant strains used in this study. Images were taken at the steady-state growth phase (day 6 in (a)). Scale bars, 10 μm.

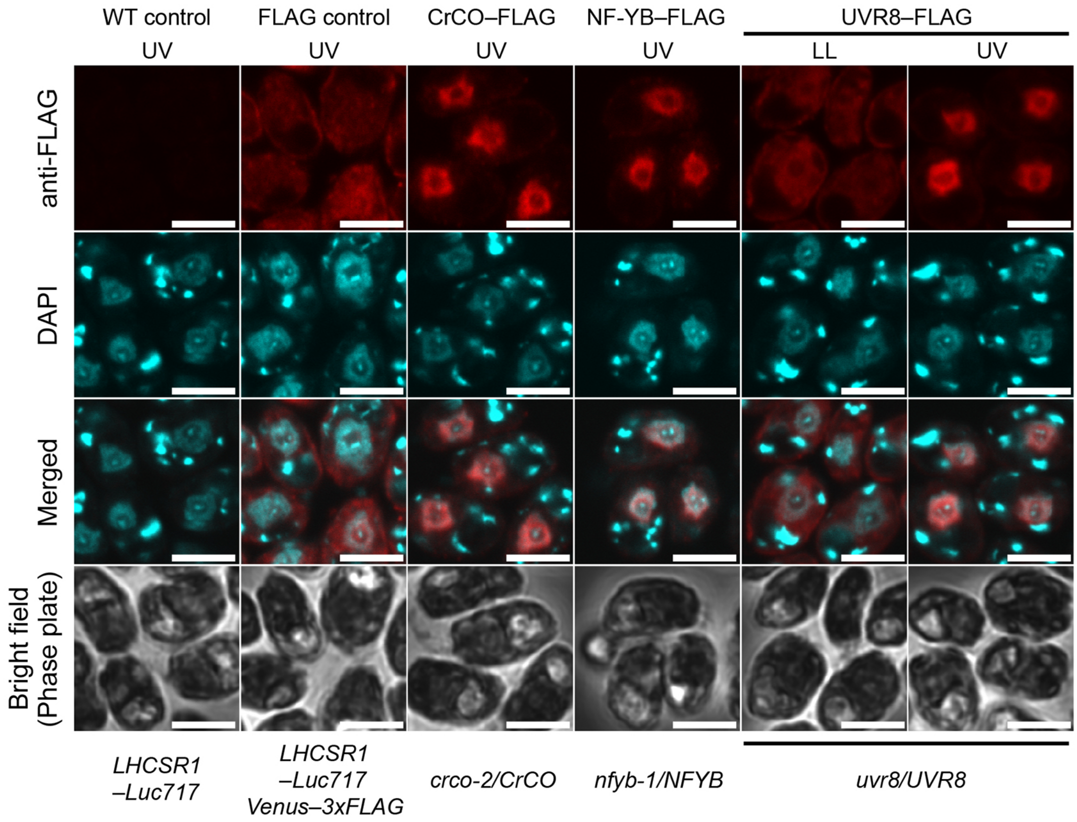

Supplementary Figure 5. Localizations of CONSTANS, NF-YB, and UVR8 under UV illumination.

Immunocytochemistry of the *C. reinhardtii* strains used in this study, *LHCSR1–Luc717* (WT control), *LHCSR1–Luc717* transformed with the Venus–3xFLAG construct (FLAG control), *crco-2/CrCO* (CrCO–FLAG), *nfyb-1/NFYB* (NF-YB–FLAG), and *uvr8/UVR8* (UVR8–FLAG), to visualize the subcellular localization of each protein. Cells were harvested after 1 h of UV treatment (and also after a 1 h LL treatment in the case of *uvr8/UVR8*) and subjected to immunocytochemistry with anti-FLAG antibody and DAPI staining. Fluorescence from AlexaFluor546 (anti-FLAG), DAPI, and their merged images are shown. Bright field images obtained with a phase plate are shown for visualizing the *Chlamydomonas* cell shape. Scale bars, 5 μm.

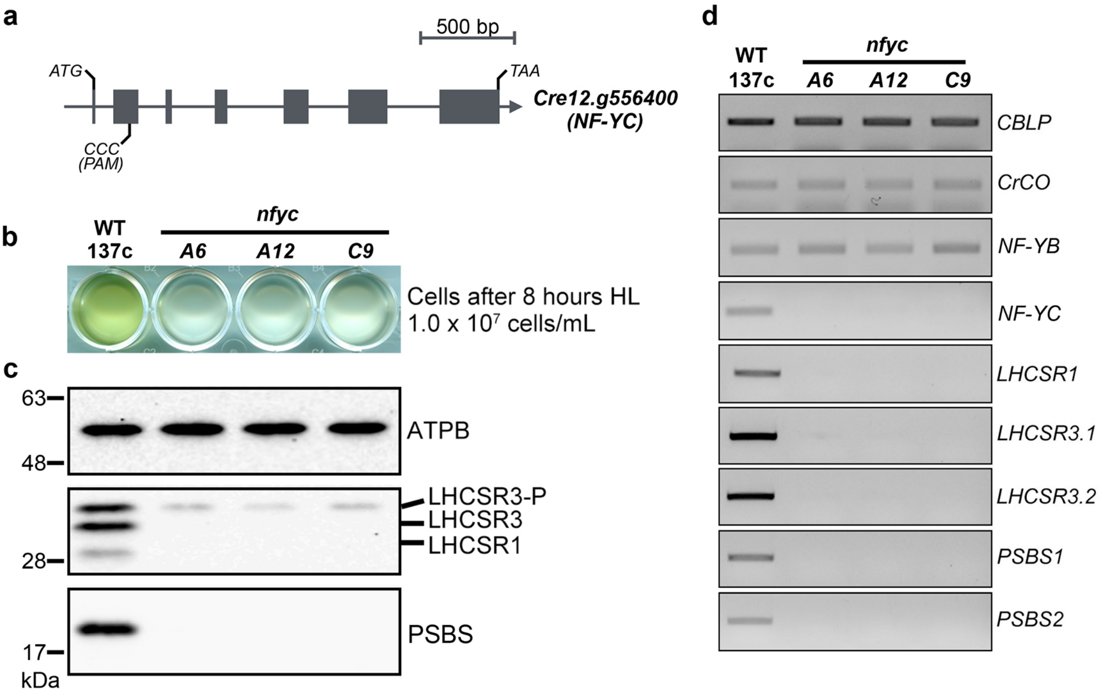

Supplementary Figure 6. Destruction of the *NF-YC* gene by the CRISPR-Cas9 system and its effects on the photoprotective response.

(a) Schematic diagram of the gene structure (chromosome) of *NUCLEAR TRANSCRIPTION FACTOR YC* (*Cre12.g556400*, *NF-YC*). The translational start (ATG) and stop (TAA) codons and the position of the PAM sequence in the mutants used in this study are shown. (b) The bleaching phenotype of the WT (137c, mt+) and NF-YC mutants (*nfyc*) was visualized in a multiwell plate. Representative cell cultures treated with HL for 8 h are shown. The concentration of cultures was adjusted to 1.0×10^7 cells/mL. (c) Immunoblot analysis of LHCSRs and PSBS after 1 h of HL treatment. ATPB proteins were included as loading controls. (d) RT-PCR analysis of UV-inducible photoprotective genes (*LHCSR1*, *LHCSR3.1*, *LHCSR3.2*, *PSBS1*, and *PSBS2*) in WT (137c) and NF-YC mutants (*nfyc*) after 1 h of HL treatment. *CBLP* signals are shown as the loading control. Representative samples of an experiment replicated three times on different biological samples are shown.

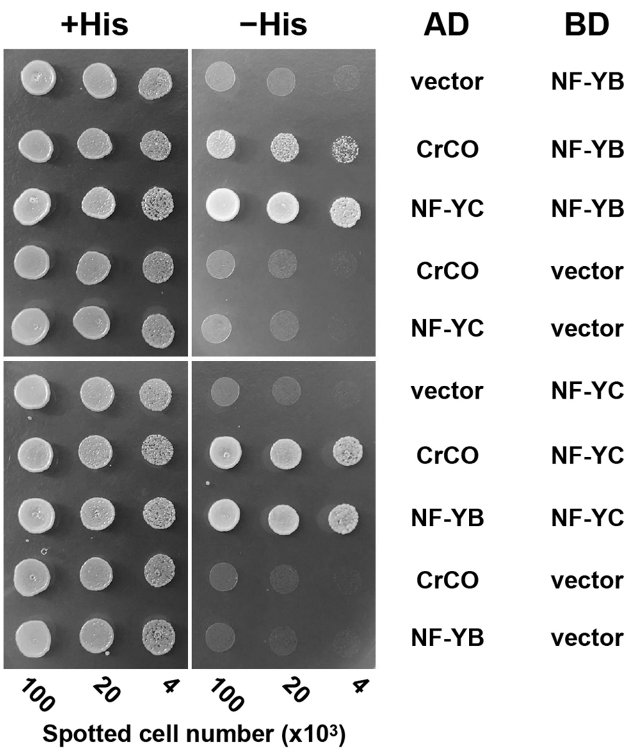

Supplementary Figure 7. Interaction among CrCO, NF-YB, and NF-YC in yeast two-hybrid (Y2H) assays.

Comprehensive Y2H assays were performed using CrCO, NF-YB, and NF-YC fused with the AD and/or BD domains of GAL4. Culture conditions were identical to those used in Fig. 2b.

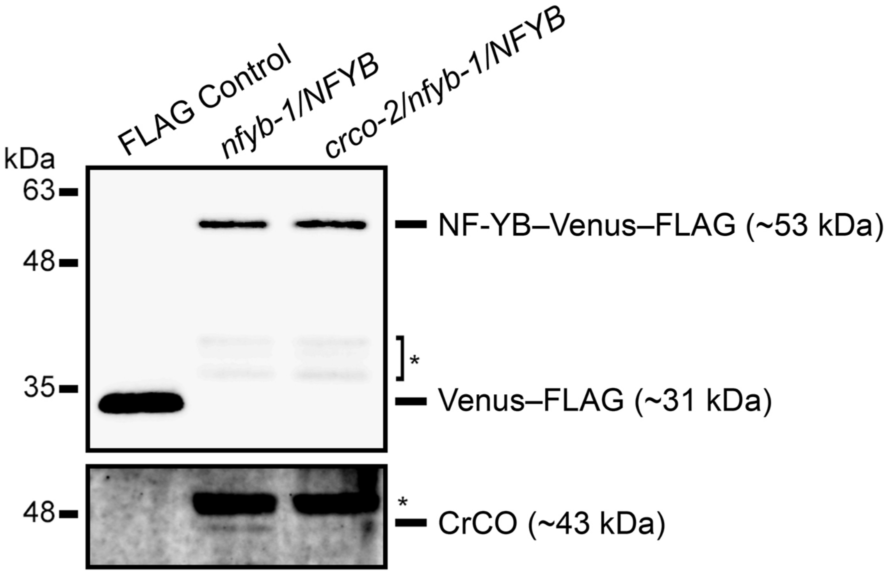

Supplementary Figure 8. Immunoblot analysis of FLAG and CrCO proteins in coimmunoprecipitated samples of NF-YB–Venus–3xFLAG.

The *LHCSR1–Luc717* strain transformed with the Venus–3xFLAG construct (FLAG control), *nfyb-1/NFYB*, and *crco-2/nfyb-1/NFYB* strains were treated with UV for 2 h. The UV-treated cells were harvested and Venus–3xFLAG or NFYB–Venus–3xFLAG proteins were immunoprecipitated by FLAG (M2) antibody with SureBeads. The CrCO protein was detected by CrCO antibody. FLAG fusion proteins were detected by FLAG (M2) antibody. Venus–3xFLAG protein (Venus–FLAG) is shown as a loading control. * indicates non-specific bands.

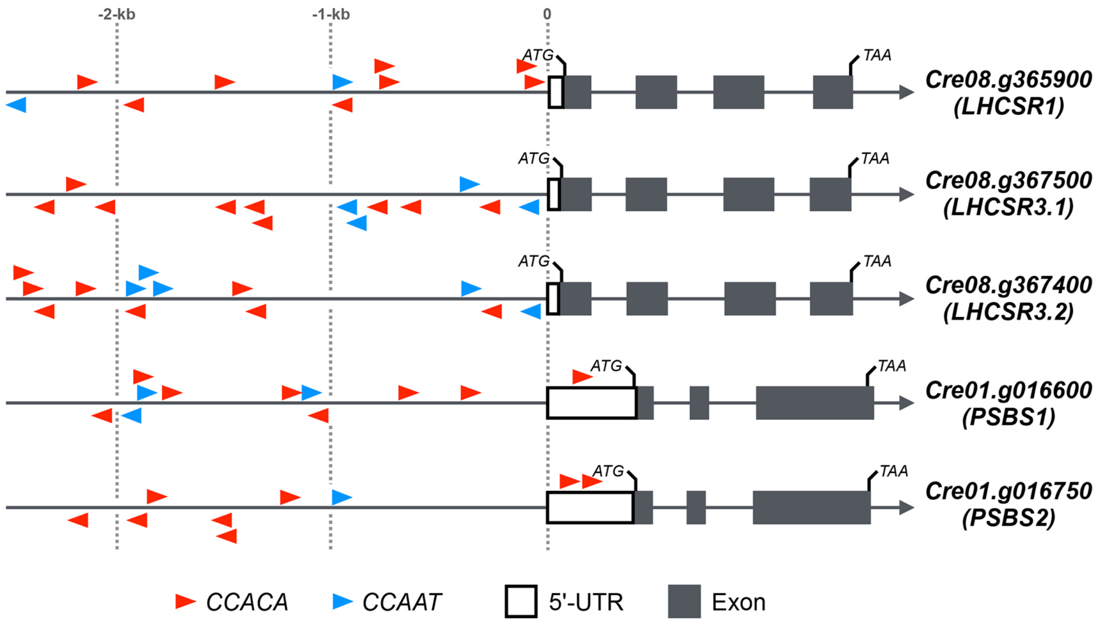

Supplementary Figure 9. Conserved responsible *cis*-elements for CONSTANS and NF-Y in the photoprotective genes in *C. reinhardtii*.

Schematic diagrams of the gene structures (chromosomes), including the upstream region (~2.5 kb), of *LHCSR1*, *LHCSR3.1 LHCSR3.2*, *PSBS1*, and *PSBS2*. Translation start (ATG) and stop (TAA) codons, the position of the 5'-UTR and exons, and responsible *cis*-elements for CONSTANS (*red arrowhead*, *CCACA*) and NF-Y (*blue arrowhead*, *CCAAT*) are shown.

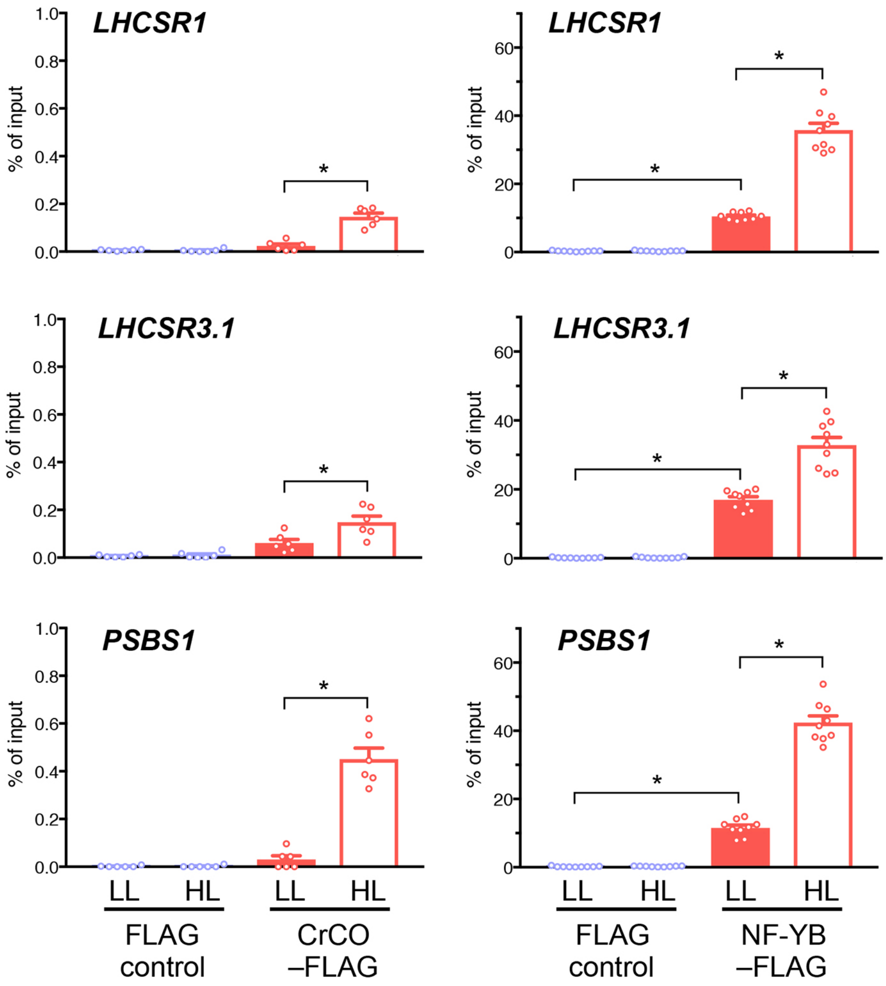

Supplementary Figure 10. Quantitative PCR assays of the photoprotective genes using ChIP samples from different light conditions.

ChIP was performed on 2 × 10^8 cells of *crco-2*/*CrCO* (labeled as CrCO–FLAG) or *nfyb-1*/*NFYB* (labeled as NF-YB–FLAG) cross-linked with 0.35% formaldehyde after 1 h of light treatment. n = 6 or n = 9 for CrCO–FLAG or NF-YB–FLAG, respectively (mean ± s.e.m.). Statistical significance was analyzed by one-way ANOVA followed by Tukey's multiple comparison test; * denotes adjusted *P* value of <0.05.

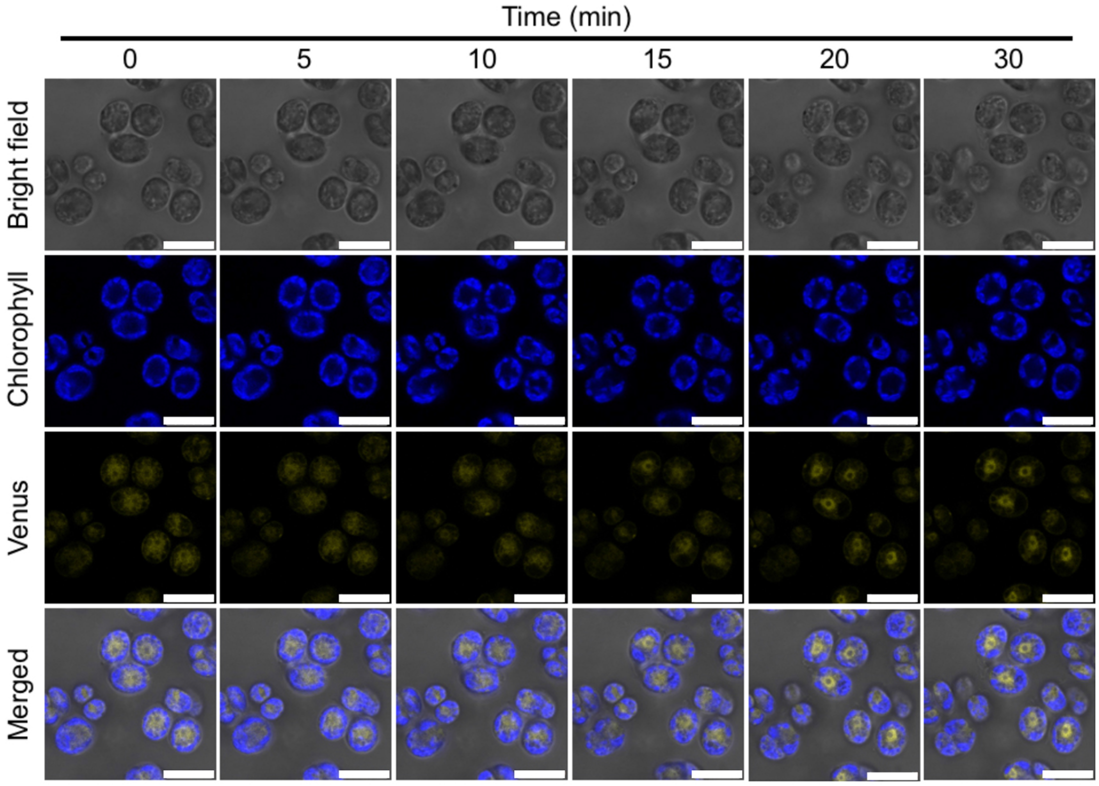

Supplementary Figure 11. UVR8-Venus accumulation around the nucleus under UV.
Time-lapse live-cell confocal imaging of UVR8–Venus–3xFLAG proteins in the *DSR1–comp15* (*uvr8*/*UVR8*) cells shown in Fig. 3a. Cells were treated with UV for a total of 30 min. Scale bars, 5 μm.

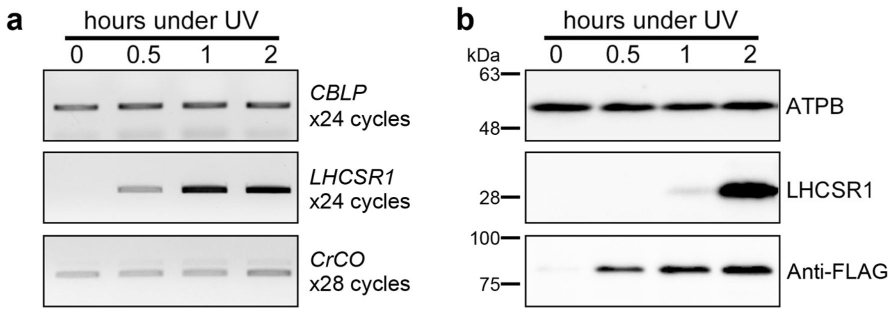

Supplementary Figure 12. ***CrCO*** **over-expression and UV-inducible CrCO accumulation in the** ***crco-2*****/*****CrCO*** **strain.**

(a) RT-PCR and (b) immunoblot analysis of *LHCSR1* and *CrCO–Venus–3xFLAG* expression during UV treatment of the *crco-2*/*CrCO*. *CBLP* was used as a housekeeping control. The CrCO–Venus–3xFLAG protein was detected by FLAG (M2) antibody. ATPB proteins are shown as loading controls.

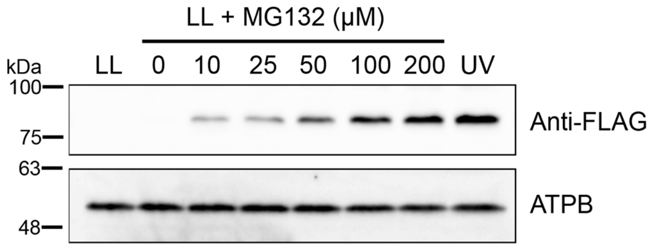

Supplementary Figure 13. Effects of a proteasome inhibitor MG132 on CrCO accumulation under LL.

Immunoblot analysis of CrCO–Venus–3xFLAG protein after a proteasome inhibitor treatment. The *crco-2*/*CrCO* cells were treated with a proteasome inhibitor MG132 at different concentrations as indicated. DMSO was used as the solvent control (0 μM MG132). The cells were cultured under LL for 2 h. Samples treated with UV for 2 h in the absence of MG132 are included as a positive control (UV). The CrCO–Venus–3xFLAG protein was detected by FLAG (M2) antibody. ATPB proteins are shown as loading controls.

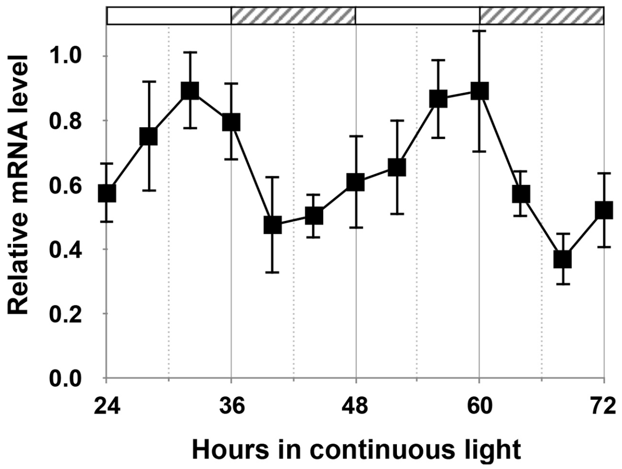

Supplementary Figure 14. Circadian mRNA rhythm of *CrCO*.

Asynchronous HS liquid cultures of the WT (CBR) strain were exposed to 12 h of darkness at 17°C to synchronize the circadian clock and then released into continuous light. Experiments were performed three times independently. Data are the mean ± s.d. of expression levels relative to the peak level (set to 1) of each experiment. The open and hatched boxes above the graph represent the subjective day and night, respectively.

Position	*m/z (Observed)*	*Mr (expt)*	*Mr (calc)*	*Delta*	*Score*	*Peptide + Modifications*
74-103	1162.6160	3484.8261	3484.8203	0.0000	57	R.KTINGDDLLWAMTTLGFEEYLEPLKLYLAK.F
74-103	872.2139	3484.8264	3484.8203	0.0042	31	R.KTINGDDLLWAMTTLGFEEYLEPLKLYLAK.F
75-103	1679.3696	3356.7247	3356.7254	0.0004	36	K.TINGDDLLWAMTTLGFEEYLEPLKLYLAK.F
75-103	1119.9170	3356.7291	3356.7254	0.0000	75	K.TINGDDLLWAMTTLGFEEYLEPLKLYLAK.F
75-103	1125.2487	3372.7242	3372.7203	0.0000	58	K.TINGDDLLWAMTTLGFEEYLEPLKLYLAK.F + Oxidation (M)

Supplementary Table 1. Unique polypeptides of NF-YB (*Cre02. g079200*) identified by LC-MS/MS analysis of in-gel trypsin digestion of coimmunoprecipitated samples of CrCO–Venus–3xFLAG after UV treatment.

The position (*Position*), experimentally observed m/z values (*m/z (Observed)*), monoisotopic mass (*Mr(*expt*)*), theoretically calculated (*Mr(calc)*) values, observed difference (*Delta*), score (*Score*), and sequence (*Peptide + Modifications*) of each observed tryptic peptide are shown.

Position	*m/z (Observed)*	*Mr (expt)*	*Mr (calc)*	*Delta*	*Score*	*Peptide + Modifications*
28-48	1202.1165	2402.2183	2402.2158	0.0025	27	K.SFWQAQLVEVSEVPPDPTVFK.N
28-56	833.9401	3331.7314	3331.7354	-0.0040	21	K.SFWQAQLVEVSEVPPDPTVFKNHQLPLAR.I
28-56	833.9418	3331.738	3331.7354	0.0026	21	K.SFWQAQLVEVSEVPPDPTVFKNHQLPLAR.I
28-56	1111.587	3331.7393	3331.7354	0.0039	67	K.SFWQAQLVEVSEVPPDPTVFKNHQLPLAR.I
49-56	474.7722	947.5298	947.5301	-0.0003	25	K.NHQLPLAR.I
59-68	407.5483	1219.623	1219.623	0.0000	41	K.KIMKSDEDVR.M
59-68	610.8195	1219.6244	1219.623	0.0014	74	K.KIMKSDEDVR.M
59-68	412.8799	1235.618	1235.618	0.0000	27	K.KIMKSDEDVR.M + Oxidation (M)
59-68	618.8163	1235.618	1235.618	0.0000	47	K.KIMKSDEDVR.M + Oxidation (M)
60-68	364.8497	1091.5272	1091.5281	-0.0009	40	K.IMKSDEDVR.M
60-68	370.1815	1107.5227	1107.523	-0.0003	46	K.IMKSDEDVR.M + Oxidation (M)
60-68	554.7691	1107.5236	1107.523	0.0006	55	K.IMKSDEDVR.M + Oxidation (M)
60-80	784.0774	2349.2103	2349.2072	0.0031	47	K.IMKSDEDVRMISAEAPVLFAK.A
60-80	592.3077	2365.2019	2365.2021	-0.0002	28	K.IMKSDEDVRMISAEAPVLFAK.A + Oxidation (M)
60-80	789.4091	2365.2055	2365.2021	0.0034	46	K.IMKSDEDVRMISAEAPVLFAK.A + Oxidation (M)
60-80	794.7397	2381.1972	2381.197	0.0002	37	K.IMKSDEDVRMISAEAPVLFAK.A + 2 Oxidation (M)
63-80	660.0037	1976.9893	1976.9877	0.0016	60	K.SDEDVRMISAEAPVLFAK.A
63-80	989.5019	1976.9893	1976.9877	0.0016	73	K.SDEDVRMISAEAPVLFAK.A
63-80	665.335	1992.9832	1992.9826	0.0006	64	K.SDEDVRMISAEAPVLFAK.A + Oxidation (M)
63-80	997.5004	1992.9863	1992.9826	0.0037	64	K.SDEDVRMISAEAPVLFAK.A + Oxidation (M)
69-80	638.8519	1275.6892	1275.6897	-0.0005	24	R.MISAEAPVLFAK.A
69-80	638.8528	1275.691	1275.6897	0.0013	64	R.MISAEAPVLFAK.A
69-80	638.8529	1275.6912	1275.6897	0.0015	49	R.MISAEAPVLFAK.A
69-80	646.85	1291.6855	1291.6846	0.0009	72	R.MISAEAPVLFAK.A + Oxidation (M)
81-92	748.3874	1494.7602	1494.7574	0.0028	78	K.ACEMFILELTLR.S
93-101	566.2483	1130.4821	1130.4815	0.0006	32	R.SWMHAEENK.R
93-102	429.8679	1286.5819	1286.5826	-0.0007	40	R.SWMHAEENKR.R
93-102	644.299	1286.5835	1286.5826	0.0009	48	R.SWMHAEENKR.R
93-102	435.2	1302.5782	1302.5775	0.0007	28	R.SWMHAEENKR.R + Oxidation (M)
93-103	481.902	1442.6841	1442.6837	0.0004	28	R.SWMHAEENKRR.T
93-103	722.3497	1442.6848	1442.6837	0.0011	32	R.SWMHAEENKRR.T
93-103	487.2335	1458.6787	1458.6786	0.0001	22	R.SWMHAEENKRR.T + Oxidation (M)
104-116	467.6	1399.7781	1399.7783	-0.0002	51	R.TLQRNDVAAAITK.T
104-116	700.8966	1399.7787	1399.7783	0.0004	55	R.TLQRNDVAAAITK.T
108-116	451.7505	901.4864	901.4869	-0.0005	54	R.NDVAAAITK.T

Supplementary Table 2. Unique polypeptides of NF-YC (*Cre12.g556400*) identified by LC-MS/MS analysis of in-gel trypsin digestion of coimmunoprecipitated samples of NF-YB–Venus–3xFLAG after UV treatment.

The position (*Position*), experimentally observed m/z values (*m/z (Observed)*), monoisotopic mass (*Mr(expt)*), theoretically calculated (*Mr(calc)*) values, observed difference (*Delta*), score (*Score*), and sequence (*Peptide + Modifications*) of each observed tryptic peptide are shown.

Position	*m/z (Observed)*	*Mr (expt)*	*Mr (calc)*	*Delta*	*Score*	*Peptide + Modifications*
64-86	885.7504	2654.2295	2654.2257	0.0038	34	K.NSCPSCGAYLTADHIYPNFLLNK.I
90-108	653.6890	1958.0453	1958.0433	0.0020	47	R.KAATATAPTSTSLLDQVQR.L
91-108	915.9825	1829.950	1829.9483	0.0021	94	K.AATATAPTSTSLLDQVQR.L
171-183	666.856	1331.6982	1331.6980	0.0002	71	R.RADSLISGLMGGR.G
171-183	450.2379	1347.6919	1347.6929	-0.0010	33	R.RADSLISGLMGGR.G + Oxidation (M)
348-362	554.2667	1659.778	1659.777	0.0010	47	R.KEAEAEEESPAMLAR.S
349-362	774.8477	1547.6808	1547.677	0.0035	71	K.EAEAEEESPAMLAR.S + Oxidation (M)
363-370	475.7382	949.462	949.462	0.0001	61	R.SSSSWLQR.A
472-503	856.0673	2565.180	2565.177	0.0028	19	R.AGSAGGGGGGRTMSGGSVTMSGAAAGGVAGGR.G
483-503	891.4187	1780.8228	1780.8196	0.0032	69	R.TMSGGSVTMSGAAAGGVAGGR.G
483-505	665.6552	1993.9436	1993.942	0.0014	38	R.TMSGGSVTMSGAAAGGVAGGRGR.V
504-521	577.9436	1730.8090	1730.807	0.0019	62	R.GRVADEEADGESVGVGGK.G
504-525	710.3584	2128.0534	2128.0509	0.0025	54	R.GRVADEEADGESVGVGGKGALR.V
506-521	759.8505	1517.6864	1517.6846	0.0018	98	R.VADEEADGESVGVGGK.G
506-525	958.4719	1914.9292	1914.9283	0.0009	77	R.VADEEADGESVGVGGKGALR.V
576-588	725.8597	1449.7049	1449.7034	0.0015	96	R.CGSADNFQLLLGR.S
664-685	945.036	1888.0573	1888.0530	0.0043	84	R.AQPKPAAVAAAATAAPGAGLGK.R
664-686	682.3927	2044.1563	2044.1541	0.0022	45	R.AQPKPAAVAAAATAAPGAGLGKR.R
664-687	551.0704	2200.253	2200.2552	-0.0026	37	R.AQPKPAAVAAAATAAPGAGLGKRR.T
688-704	666.92	1997.7403	1997.7392	0.0011	75	R.TRDEDTDADDEDEMDAR.E
688-706	761.9689	2282.8850	2282.8829	0.0021	68	R.TRDEDTDADDEDEMDARER.R
688-706	767.3008	2298.8805	2298.8778	0.0027	65	R.TRDEDTDADDEDEMDARER.R + Oxidation (M)
711-739	951.8057	2852.3954	2852.3875	0.0079	47	R.GAAGAANGTVQLPPYAHTTAAMQPSQQSK.Q
711-739	957.1354	2868.3845	2868.3824	0.0021	36	R.GAAGAANGTVQLPPYAHTTAAMQPSQQSK.Q + Oxidation (M)
783-793	498.7833	995.5520	995.5512	0.0008	60	R.APAQAAIAAGR.R
783-793	384.8912	1151.6518	1151.6523	-0.0005	33	R.APAQAAIAAGRR.D
795-812	718.9472	2153.8198	2153.8178	0.0020	69	R.DKDKEEDEDGEEDEMDAR.E
795-812	543.4606	2169.8134	2169.8128	0.0006	25	R.DKDKEEDEDGEEDEMDAR.E + Oxidation (M)
880-889	619.3165	1236.6185	1236.6172	0.0013	56	R.MLSQFDVLER.C
880-895	697.0062	2087.9967	2087.9955	0.0012	34	R.MLSQFDVLERCYLQMR.A
880-895	702.3381	2103.9924	2103.9904	0.0020	39	R.MLSQFDVLERCYLQMR.A + Oxidation (M)
880-895	707.6700	2119.9881	2119.9853	0.0028	38	R.MLSQFDVLERCYLQMR.A + 2 Oxidation (M)
1027-1035	507.294	1012.5741	1012.5739	0.0002	50	R.LEMLAAIPR.Y
1036-1049	450.6	1348.6484	1348.6484	0.0000	55	R.YGSERSGPGAGGVR.G
1093-1109	634.348	1900.0217	1900.0206	0.0011	62	R.ILVYDYQALLAAAPHSR.G
1110-1141	682.1057	2724.3937	2724.3903	0.0034	39	R.GGGGGGGGGDGSGSAGAIAAARPVLELTARTK.M
1269-1275	411.7451	821.4756	821.4759	-0.0003	34	R.KAVSYVR.W
1337-1344	401.7	801.4345	801.4344	0.0001	50	R.SAGAELVR.T
1337-1353	631.3076	1890.9010	1890.9006	0.0004	29	R.SAGAELVRTFTGHMNER.N + Oxidation (M)
1345-1353	554.7455	1107.4765	1107.4767	-0.0002	42	R.TFTGHMNER.N + Oxidation (M)

Supplementary Table 3. Unique polypeptides of COP1 (*Cre02.g085050*) identified by LC-MS/MS analysis of in-gel trypsin digestion of coimmunoprecipitated samples of UVR8–Venus–3xFLAG after UV treatment.

The position (*Position*), experimentally observed m/z values (*m*/z *(Observed)*), monoisotopic mass (*Mr(expt)*), theoretically calculated (*Mr(calc)*) values, observed difference (*Delta*), score (*Score*), and sequence (*Peptide + Modifications*) of each observed tryptic peptide are shown.

Position	*m/z (Observed)*	*Mr (expt)*	*Mr (calc)*	*Delta*	*Score*	*Peptide + Modifications*
6-13	442.2901	882.5656	882.5651	0.0005	20	R.LVVGNLLR.A
17-34	646.3586	1936.0539	1936.0517	0.0022	57	K.YKVTIAESGTQALEVLSK.A
193-243	826.1713	4125.820	4125.8115	0.0088	15	R.AAALAASTTAHHHPGAGGGSGSGGGAMDAGGGGAGGGGGGGGGLPDDAAMR.L
193-243	1036.459	4141.8054	4141.8064	-0.0010	31	R.AAALAASTTAHHHPGAGGGSGSGGGAMDAGGGGAGGGGGGGGGLPDDAAMR.L + Oxidation (M)
193-243	1036.4608	4141.8142	4141.8064	0.0078	25	R.AAALAASTTAHHHPGAGGGSGSGGGAMDAGGGGAGGGGGGGGGLPDDAAMR.L + Oxidation (M)
391-414	774.7459	2321.216	2321.213	0.0031	68	R.VAFVQPPPSAAAAATAGQQQQVGK.A
391-414	1161.6160	2321.2174	2321.213	0.0046	68	R.VAFVQPPPSAAAAATAGQQQQVGK.A
528-535	479.2779	956.541	956.540	0.0010	33	R.AELLREAR.Q
536-545	390.8964	1169.667	1169.667	0.0005	30	R.QRVLPPAFSR.S
588-632	1124.9060	3371.6962	3371.6930	0.0032	101	R.AAAAAAAAAAGAVAAGGAGALGGEAAGQQGGAGAGAGVAPGGAAK.R
588-632	843.9317	3371.6977	3371.693	0.0047	83	R.AAAAAAAAAAGAVAAGGAGALGGEAAGQQGGAGAGAGVAPGGAAK.R
588-633	882.9569	3527.7983	3527.794	0.0042	91	R.AAAAAAAAAAGAVAAGGAGALGGEAAGQQGGAGAGAGVAPGGAAKR.A
588-633	1176.9408	3527.8006	3527.7941	0.0065	85	R.AAAAAAAAAAGAVAAGGAGALGGEAAGQQGGAGAGAGVAPGGAAKR.A
924-945	648.3347	1941.9822	1941.9769	0.0053	66	R.RGSTAAAVAASAAGSGAAGWPR.G
924-945	596.2993	1785.8760	1785.8758	0.0002	36	R.GSTAAAVAASAAGSGAAGWPR.G
924-945	893.9464	1785.8783	1785.876	0.0025	98	R.GSTAAAVAASAAGSGAAGWPR.G
925-963	1135.5617	3403.6631	3403.6539	0.0092	24	R.GSTAAAVAASAAGSGAAGWPRGGSMSGTTASGVVATTPR.H
946-963	818.9029	1635.7912	1635.7887	0.0025	104	R.GGSMSGTTASGVVATTPR.H
946-963	826.900	1651.7849	1651.7836	0.0013	97	R.GGSMSGTTASGVVATTPR.H + Oxidation (M)
966-978	673.3649	1344.7152	1344.7150	0.0002	62	R.HADAGGLPEPLLR.F
1178-1187	364.1904	1089.5494	1089.5502	-0.0008	27	R.ALHVMPGHGR.A + Oxidation (M)
1225-1236	502.2407	1503.700	1503.7001	0.0002	26	R.CVQTFRGHTNER.N
1237-1257	1174.0084	2346.0023	2346.0005	0.0018	67	R.NFTGLSVSPDGYICCGSENNR.V

Supplementary Table 4. Unique polypeptides of SPA1 (*Cre13.g602700*) identified by LC-MS/MS analysis of in-gel trypsin digestion of coimmunoprecipitated samples of UVR8–Venus–3xFLAG after UV treatment.

The position (*Position*), experimentally observed m/z values (*m/z (Observed)*), monoisotopic mass (*Mr(expt)*), theoretically calculated (*Mr(calc)*) values, observed difference (*Delta*), score (*Score*), and sequence (*Peptide + Modifications*) of each observed tryptic peptide are shown.

Position	*m/z (Observed)*	*Mr (expt)*	*Mr (calc)*	*Delta*	*Score*	*Peptide + Modifications*
6-30	646.3586	2477.3345	2477.2545	0.0800	45	K.TLTGKTITLEVESSDTIENVKAK.I
6-28	826.1713	2278.2024	2278.1828	0.0197	59	K.TLTGKTITLEVESSDTIENVK.A
11-28	1036.4586	1777.907	1777.8116	0.0950	37	K.TITLEVESSDTIENVK.A
29-43	1174.008	1523.7813	1523.7598	0.0215	46	K.IQDKEGIPPDQQR.L
48-73	442.2901	2812.4952	2812.4484	0.0468	20	K.QLEDGRTLADYNIQKESTLHLVLR.L

Supplementary Table 5. Unique polypeptides of Ubiquitin (*Cre09.g396400*) identified by LC-MS/MS analysis of in-gel trypsin digestion of coimmunoprecipitated samples of CrCO–Venus–3xFLAG.

The position (*Position*), experimentally observed m/z values (*m/z (Observed)*), monoisotopic mass (*Mr(expt)*), theoretically calculated (*Mr(calc)*) values, observed difference (*Delta*), score (*Score*), and sequence (*Peptide + Modifications*) of each observed tryptic peptide are shown.

Primer name	*Sequences (5′ to 3′)*
DegPstI	*CCAGTGAGCAGAGTGACGIIIIINNSCTGCAGW*
DegSacII	*CCAGTGAGCAGAGTGACGIIIIINNSCCGCGGW*
DegBglII	*CCAGTGAGCAGAGTGACGIIIIINNSAGATCTS*
DegMluI	*CCAGTGAGCAGAGTGACGIIIIINNSACGCGTW*
Q0	*CCAGTGAGCAGAGTGACG*
aph7tag-F1	*GACGTCTATGCGGGAGACTC*
aph7tag-F2	*CTTCGAGGTGTTCGAGGAGACC*
aph7tag-F3	*GTAAATGGAGGCGCTCGTTGATC*
aph7tag-R1	*AGCGGCTGCAAATGGAAACG*
aph7tag-R2	*GATGCTGCTTGAGACAGCGAC*
aph7tag-R3	*CTCCCAGAATTCCTGGTCGTTC*
DSR28-EcoRI-F	*TTTTGAATTCGGCTTCTCCGCGATGAG*
DSR28-R2	*CTTTGCCTCCGCGTTGGCAC*
DSR28-F2	*TTGGGCTTTGAGGAGTACCTG*
Cre12(2898864R)	*GCGTGTCGGTTCCCATGGAC*
Cre12(2894770F)	*ACGTCCGGCTGCTCCGATTC*
HDR-F	*ACAGCTGGTCGAGGTGTCTGAGGTCCCACCTTGATAGACCACGAC ATCGACTACAAGGACTGACCCAACTGTATTCAAGGTATGGCATGT*
HDR-R	*ACATGCCATACCTTGAATACAGTTGGGTCAGTCCTTGTAGTCGATGT CGTGGTCTATCAAGGTGGGACCTCAGACACCTCGACCAGCTGT*
NFYC-check-F	*CCTCTCCTGAACTCTTTGACTG*
FLAG-R	*CCTTGTAGTCGATGTCGTGGTC*

Supplementary Table 6. Primers described in Methods.

Primer name	Sequences (5′ to 3′)	Amplicon size
CBLP (top strand)	*AGGTCTGGAACCTGACCAACT*	*199 bp*
CBLP (bottom strand)	*AAGCACAGGCAGTGGATGA*	
LHCSR1 (top strand)	*TGTTGGCAGAATTGTGTGACATGG*	*412 bp*
LHCSR1 (bottom strand)	*GCCCATTTCTTATACATCCGATGCAC*	
LHCSR3.1 (top strand)	*GCTTGTTCCCGCTCGAGC*	*441 bp*
LHCSR3.1 (bottom strand)	*GCTCCGTGGAGCCTGCTC*	
LHCSR3.2 (top strand)	*CCGCTTGCTTCTGCTCAAGTTC*	*530 bp*
LHCSR3.2 (bottom strand)	*CTCTCGCCTGTTGTCACCATC*	
PSBS1 (top strand)	*AGGGTAGAACAGCTATGGTTTCGT*	*331 bp*
PSBS1 (bottom strand)	*CCGTCAGATCCCGTTCTCTCTG*	
PSBS2 (top strand)	*CAGGGTAGAACAGCTATGGTTTCAG*	*312 bp*
PSBS2 (bottom strand)	*GCGTTGTACGTCTTGCGTCAC*	
LHCSR1 5'UTR (top strand)	*CAGACAGCGCTTTGAGGAGC*	*318 bp*
LHCSR1 5'UTR (bottom strand)	*GGTCGTACGTGTCGGTTGTG*	
LHCSR3.1 5'UTR (top strand)	*GCATCCTGCGATCCCGAACA*	*308 bp*
LHCSR3.1 5'UTR (bottom strand)	*GAACAAGATGGGCTATTGACAGC*	
PSBS1 5'UTR (top strand)	*GGTCGCGGGCACATATTCAC*	*303 bp*
PSBS1 5'UTR (bottom strand)	*CTGGTGCCGTACAGGAATTCG*	
CrCO (top strand)	*AACGCGGTCGCTGCGCGCCATAC*	*187 bp*
CrCO (bottom strand)	*GTGCAGCGCCCTGCTCGACTGAC*	
NF-YB (top strand)	*GTTCAGAGAGGCTGAGGCGG*	*218 bp*
NF-YB (bottom strand)	*GCGGTAGCCCCTGGAACTG*	
NF-YC (top strand)	*CCTCTGGCCCGCATCAAAAAG*	*115 bp*
NF-YC (bottom strand)	*GCGTCAGCTCCAGGATGAAC*	
RCK1 (top strand)	*GTGGACAACCGCCAGATCGTGTCG*	*196 bp*
RCK1 (bottom strand)	*GGTTCCAGACCTTGACCATCTTGTCCC*	

Supplementary Table 7. Primers used for the RT-PCR, ChIP-qPCR, and circadian mRNA rhythm analyses.

Primer name	*Sequences (5' to 3')*	*Amplicon size*
CrCO-EcoRI-F	*TATAGAATTCATGTCGAGTTGCGTCGTGT*	*911 bp*
CrCO-SalI-R	*TTTTGTCGACTTAGCACTCAGCGTCCAG*	
NF-YB-EcoRI-F	*TTTTGAATTCGGCTTCTCCGCGATGAG*	*1253 bp*
NF-YB-SalI-R	*TTTTGTCGACGTAAGATTGCAGCCCGCTA*	
NF-YC-EcoRI-F	*TATAGAATTCATGGGGGACCAGTATAACT*	*678 bp*
NF-YC-SalI-R	*TTTTGTCGACTTACTCCTGCTTTGCAGCC*	
COP1-F-Y2H	*CGGAATTCCCGGGGATCATGTCAGTCAC*	*4170 bp*
COP1-R-Y2H	*ATAGATCTCTGCAGGTCGACTACAGCTG*	
SPA1-F-Y2H	*CGGAATTCCCGGGGATCATGCGCCTCAC*	*4368 bp*
SPA1-R-Y2H	*ATAGATCTCTGCAGGTCGATCAGCTGAG*	

Supplementary Table 8. Primers for Y2H construction described in Methods.
