# Supplementary material for: The CONSTANS flowering complex controls the protective response of photosynthesis in the green alga Chlamydomonas
Source: Nat Commun. 2019 Sep 10;10:4099. doi: 10.1038/s41467-019-11989-x (PMC6736836; doi:10.1038/s41467-019-11989-x)
Supplement: Supplementary file 3 — Reporting Summary [file 41467_2019_11989_MOESM3_ESM.pdf]

nature research

Corresponding author(s): Ryutaro Tokutsu / Jun Minagawa

Last updated by author(s): Aug 6, 2019

Reporting Summary

Nature Research wishes to improve the reproducibility of the work that we publish. This form provides structure for consistency and transparency in reporting. For further information on Nature Research policies, see Authors & Referees and the Editorial Policy Checklist .

Statistics

For all statistical analyses, confirm that the following items are present in the figure legend, table legend, main text, or Methods section.

n/a	Confirmed	
☐	☒	The exact sample size (*n*) for each experimental group/condition, given as a discrete number and unit of measurement
☐	☒	A statement on whether measurements were taken from distinct samples or whether the same sample was measured repeatedly
☐	☒	The statistical test(s) used AND whether they are one- or two-sided<br>*Only common tests should be described solely by name; describe more complex techniques in the Methods section.*
☒	☐	A description of all covariates tested
☐	☒	A description of any assumptions or corrections, such as tests of normality and adjustment for multiple comparisons
☐	☒	A full description of the statistical parameters including central tendency (e.g. means) or other basic estimates (e.g. regression coefficient) AND variation (e.g. standard deviation) or associated estimates of uncertainty (e.g. confidence intervals)
☐	☒	For null hypothesis testing, the test statistic (e.g. *F*, *t*, *r*) with confidence intervals, effect sizes, degrees of freedom and *P* value noted<br>*Give P values as exact values whenever suitable.*
☒	☐	For Bayesian analysis, information on the choice of priors and Markov chain Monte Carlo settings
☒	☐	For hierarchical and complex designs, identification of the appropriate level for tests and full reporting of outcomes
☒	☐	Estimates of effect sizes (e.g. Cohen's *d*, Pearson's *r*), indicating how they were calculated

Our web collection on statistics for biologists contains articles on many of the points above.

Software and code

Policy information about availability of computer code

Data collection

1. FluorCAM7 (Photon Systems Instruments, Czech Republic) software was used for collecting data of photosynthetic parameters.
2. Image Lab (Bio-Rad Laboratories, Hercules, CA, USA) software was used for collecting immunoblot data.
3. LASX (Leica Microsystems, Germany) software was used for collecting live-cell imaging data.
4. Light Cycler 96 system (Roche Diagnostics, Germany) was used for collecting ChIP-qPCR data.

Data analysis

1. Prizm 7 for Mac OS X (GraphPad Inc.) software was used for used for statistical analyses of photosynthetic parameters and ChIP-qPCR data.
2. Bowtie2 (bowtie-bio.sourceforge.net/bowtie2/index.shtml), SAMtools (samtools.courveforge.net/), and freebayes (https://github.com/ekg/freebayes/) softwares were used for analyzing the whole genome sequence data.
3. LASX (Leica Microsystems, Germany) software was used for analyzing the live cell images.
4. MAFFT v7.407 (https://mafft.cbrc.jp/alignment/software/), RAxML v8.2.12 (https://cme.h-its.org/exelixis/web/software/raxml/index.html), and MEGA7 (https://www.megasoftware.net/) softwares were used for the phylogenetic analysis.

For manuscripts utilizing custom algorithms or software that are central to the research but not yet described in published literature, software must be made available to editors/reviewers. We strongly encourage code deposition in a community repository (e.g. GitHub). See the Nature Research guidelines for submitting code & software for further information.

Data

Policy information about availability of data

All manuscripts must include a data availability statement. This statement should provide the following information, where applicable:

- Accession codes, unique identifiers, or web links for publicly available datasets
- A list of figures that have associated raw data
- A description of any restrictions on data availability

The sequencing data have been deposited in [DDBJ Sequence Read Archive (SRA) (http://www.ddbj.nig.ac.jp/dra)] with the accession codes [DRX140139

(#443-1A-12-5a), DRX140140 (#443-1A-12-5d), DRX140141 (DSR28-7c-4a), DRX140142 (DSR28-7c-4b), DRX140143 (DSR28-7c-4c), and DRX140144 (DSR28-7c-4d)].
The proteomic data that support the findings of this study are available in [Japan ProteOme STandard Repository (jPOSTrepo) (https://repository.jpostdb.org/)] with the accession codes [JPST000646 (Table S1), JPST000647 (Table S2), JPST000648 (Table S3), JPST000649 (Table S4), and JPST000650 (Table S5)]. The source data underlying Figs. 1b-e, 2c, 3b, 3d and Supplementary Figs. 2b, 3, 4a, 6c, 6d, 9, 10, 12, 13, and 14 are provided as a Source Data file.
All other data are available in the manuscript.

Field-specific reporting

Please select the one below that is the best fit for your research. If you are not sure, read the appropriate sections before making your selection.

☒ Life sciences ☐ Behavioural & social sciences ☐ Ecological, evolutionary & environmental sciences

For a reference copy of the document with all sections, see nature.com/documents/nr-reporting-summary-flat.pdf

Life sciences study design

All studies must disclose on these points even when the disclosure is negative.

Sample size	Statistical methods were not used to predetermine the sample size. The sample sizes (n) were written on the manuscript.
Data exclusions	We have not excluded any data from the analyses.
Replication	We conducted all experiments with replication at least three and with using independent biological replicates. The sample sizes (n) were written on the manuscript.
Randomization	Randomization was not considered in this work.
Blinding	The investigators were not blinded to allocation during experimental procedures and data assessment.

Reporting for specific materials, systems and methods

We require information from authors about some types of materials, experimental systems and methods used in many studies. Here, indicate whether each material, system or method listed is relevant to your study. If you are not sure if a list item applies to your research, read the appropriate section before selecting a response.

Materials & experimental systems

n/a	Involved in the study	
☐	☒	Antibodies
☒	☐	Eukaryotic cell lines
☒	☐	Palaeontology
☒	☐	Animals and other organisms
☒	☐	Human research participants
☒	☐	Clinical data

Methods

n/a	Involved in the study	
☒	☐	ChIP-seq
☒	☐	Flow cytometry
☒	☐	MRI-based neuroimaging

Antibodies

Antibodies used	Antibodies against ATPB, LHCSRs, PSBS, CrCO, and FLAG were used.
Validation	1. Rabbit polyclonal antibody against ATPB (AS05 085) was obtained from Agrisera (Sweden). Rabbit polyclonal antibodies against LHCSRs and CrCO were raised and affinity-purified against the peptide LGLKPTDPEELK and AAWFVDDEKMG, respectively (Eurofins Genomics). The PSBS antibody was kindly provided by Dr. Peter Jahns (Heinrich-Heine-University Düsseldorf, Germany). The detail of the PSBS antibody is described in Correa-Galvis et al. JBC (2016) doi: 10.1074/jbc.M116.737312. An anti-rabbit horseradish-peroxidase-conjugated antiserum (#7074, Cell Signaling Technology, Danvers, MA, USA) was used as a secondary antibody.<br>2. Mouse monoclonal antibody against FLAG (M2) was purchased from Sigma–Aldrich (St. Louis, MO, USA). An anti-mouse horseradish-peroxidase-conjugated antiserum, Anti-IgG (H+L chain) (Mouse) pAb-HRP (Cat. 330, MBL, Japan) was used as a secondary antibody for 3xFLAG immunoblotting and immunoprecipitation.<br>3.A secondary antibody [AlexaFluor546-conjugated F(ab')2 fragment of goat anti-mouse IgG; Thermo Fisher Scientific] was used for immunocytochemistry.
